# Control of Precision Grip Force in Lifting and Holding of Low-Mass Objects

**DOI:** 10.1371/journal.pone.0138506

**Published:** 2015-09-16

**Authors:** Yuichi Hiramatsu, Daisuke Kimura, Koji Kadota, Taro Ito, Hiroshi Kinoshita

**Affiliations:** 1 Department of Biomechanics and Motor Control, Graduate School of Medicine, Osaka University, Osaka, Japan; 2 Department of Health and Sports Sciences, Mukogawa Women’s University, Hyogo, Japan; Ludwig-Maximilian University, GERMANY

## Abstract

Few studies have investigated the control of grip force when manipulating an object with an extremely small mass using a precision grip, although some related information has been provided by studies conducted in an unusual microgravity environment. Grip-load force coordination was examined while healthy adults (N = 17) held a moveable instrumented apparatus with its mass changed between 6 g and 200 g in 14 steps, with its grip surface set as either sandpaper or rayon. Additional measurements of grip-force-dependent finger-surface contact area and finger skin indentation, as well as a test of weight discrimination, were also performed. For each surface condition, the static grip force was modulated in parallel with load force while holding the object of a mass above 30 g. For objects with mass smaller than 30 g, on the other hand, the parallel relationship was changed, resulting in a progressive increase in grip-to-load force (GF/LF) ratio. The rayon had a higher GF/LF force ratio across all mass levels. The proportion of safety margin in the static grip force and normalized moment-to-moment variability of the static grip force were also elevated towards the lower end of the object mass for both surfaces. These findings indicate that the strategy of grip force control for holding objects with an extremely small mass differs from that with a mass above 30 g. The data for the contact area, skin indentation, and weight discrimination suggest that a decreased level of cutaneous feedback signals from the finger pads could have played some role in a cost function in efficient grip force control with low-mass objects. The elevated grip force variability associated with signal-dependent and internal noises, and anticipated inertial force on the held object due to acceleration of the arm and hand, could also have contributed to the cost function.

## Introduction

Small objects are commonly manipulated using a precision grip with the tips of the index finger and the thumb. During the past few decades, considerable effort has been devoted by researchers in neuroscience to studying sensori-motor function involved in this simple mode of grip for lifting, holding, and transporting objects [1,2]. Researchers commonly used an instrumented movable object, aiming to evaluate finger forces normal (grip force) and tangential (lift or load force) to the grip surface, and in some cases tangential torques [3–5]. They found that the grip-to-load force balance was automatically adjusted to a given finger-surface frictional condition. The more slippery the grasping surface, the higher the ratio of grip force to load force (hereafter denoted as the GF/LF ratio). They also showed that the grip force was composed of two components: a voluntarily and/or reflexively adjusted excess force for preventing the object slipping from the fingers, and a minimum required force (slip force) that could be determined by current finger-surface friction. The excess force, namely, a grip’s safety margin force, appeared to be set relatively small in healthy young adults (generally some 20–50% of the employed grip force in healthy adults) over the range of frictional properties of the objects manipulated daily [6–12]. Further studies using microneurography demonstrated that tactile afferent signals were used primarily for the central nervous system (CNS) to adjust the safety margin [13,14]. CNS also relies on feed-forward or anticipatory control of grip and load forces for the lifting of objects. Visual information on an object’s properties, such as mass (or weight), density, and friction, and sensory memory acquired in previous lifts are usually used in this process [15–18].

The instrumented object used by the previous researchers for studying grasp stability commonly had a mass of a few hundred grams or more [5,7–9,12,18–28]. The effect of a small mass (< 100 g) of the object in normal-gravity conditions on grip-load force coordination had thus not been examined. Using a gravity-altered environment induced by parabolic flights, on the other hand, researchers have recently explored finger force coordination while manipulating an object under a weightless, normal, or double weight condition [24–28]. Hermsdorfers et al. [26] reported that the grip force on an object held stationary by two relatively naive subjects during a parabola was scaled to the weight of the object for normal- and high-gravity conditions. However, during weightlessness, although no grip force was needed to stabilize the object, the subjects exerted grip force of above 2.5 N on the object during the first flight, which progressively decreased to about 0.9 N at the fifth trial. Hermsdorfers et al. [26] stated that this excess grip force could be purely a safety margin in the event of possible perturbation. Crevecoeur et al. [25] later showed that this margin could be further reduced to less than 0.4 N after 10 consecutive parabolic flights with increased time for adaptation to the unusual microgravity.

Sensori-motor control of fingertip forces for handling low-mass objects (e.g., <50 g) in a normal-gravity environment may differ in several ways from that for objects of a few hundred grams or more that were commonly used in tests in previous studies. Firstly, the grip force needed for holding such a low-mass object should be quite weak, limiting the finger-surface contacting area, as well as the magnitude of skin deformation at the finger pad. This may also confine tactile sensations required for fine adjustment of the safety margin. Psychophysical studies of sensory thresholds also previously demonstrated that the relationship between the difference threshold for intensity and the intensity level of a stimulus (Weber’s fraction) increases at extremely low intensities of stimuli, including mass of a hand-held object [29,30], and finger force or pressure [31]. In addition, a common finding from numerous studies of reduced fingertip tactile sensibility is that individuals use a higher GF/LF ratio with an elevated safety margin than normal or necessary [7,10,12,20,32–35]. We thus conjecture that, during the manipulation of extremely light objects, individuals may have to use a strategy of finger force control that is to some extent similar to that for the condition of decreased tactile sensibility.

Secondly, a steady control of grip force at extremely low levels during holding of a light object may be more centrally demanding due to a decreased signal-to-noise ratio than those at moderate to high levels required for holding heavier objects. Studies of optimal control of motor system have shown that Fitts’ law emerges from the presence of signal-dependent noise (SDN) in motor commands, i.e. the noise whose variance scales in proportion to the magnitude of motor command [36–38]. The central idea is that motor command is chosen to minimize the negative effects or cost of SDN on task performance. The presence of SDN predicts that fluctuations in grip force during the precision grip would be proportionally change across all levels of grip force (intended motor command) generated. This, however, may not be true for small force production. Studies on isometric force production against an immovable object have demonstrated that the coefficient of variation (CV) value of generated force as an index of normalized force fluctuations (i.e. a noise-to-signal ratio), is clearly raised towards the smaller end of the level of force generated [39–43]. Thus, there seems to be an additional source of neural command that elevates motor output variability when the level of force manipulating is low. During precision-grip holding of an object with a small grip force safety margin, if a proportion of fluctuation in the grip force is increased, there can be increased risk of slipping and accidentally dropping the held object, which can also be fear for the holder. This can be avoidable by increasing a proportion of grip force safety margin, leading to a higher GF/LF ratio. Whether this would be the case during holding of a low-mass movable object remains to be examined.

Thirdly, the magnitude of slip force can also be quite small for extremely light objects. Westling and Johansson [12] reported that the slip-to-load force ratio was fairly constant for a lifted object weighing more than 100 g, following Amontons’ first law of friction. Tribological studies, on the other hand, have shown that, against a flat surface with varied materials, friction of the human skin increased non-linearly with decreased normal force typically less than 0.5 N applied to the skin [44,45]. It is thus quite reasonable to hypothesize that the GF/LF ratio can be lowered with this frictional change while manipulating lighter objects with a small grip force.

The present study was intended to test the above-mentioned hypotheses in a normal gravity environment. To examine variables of grip-load force coordination, healthy adults lifted and held a force-sensor-equipped movable object with a mass ranging from very small (6 g) to moderate (200 g) for slippery (rayon) and non-slippery (sandpaper) surface conditions. To examine the mass sensibility under the current experimental conditions, using an object with the same dimensions and grip surfaces as those of the instrumented test object, a weight discrimination test was also performed by the subjects. In addition, to facilitate the understanding of cutaneous mechanoreceptors that could be involved in the present precision grip task, changes in the finger-surface contact area and the finger pad indentation with grip force were examined.

## Materials and Methods

### Ethics Statement

All participants gave written informed consent to participate in the study, and the study was approved by the Ethics Committee of the Graduate School of Medicine, Osaka University (No. 11208) in accordance with the ethical standards established by the 1964 Declaration of Helsinki.

### Subjects

Eight females and nine males ranging in age from 18 to 30 years (mean age = 22.2 years) participated in the present study. They were all healthy and right-hand-dominant, as determined by the Edinburgh MRC Handedness Inventory [46]. None of them had a previous history of musculoskeletal or neurological problems in the upper limbs. This study was approved by the Ethics Committee of the Graduate School of Medicine, Osaka University, and informed consent was obtained from all subjects.

### Grip apparatus

**Fig 1:**
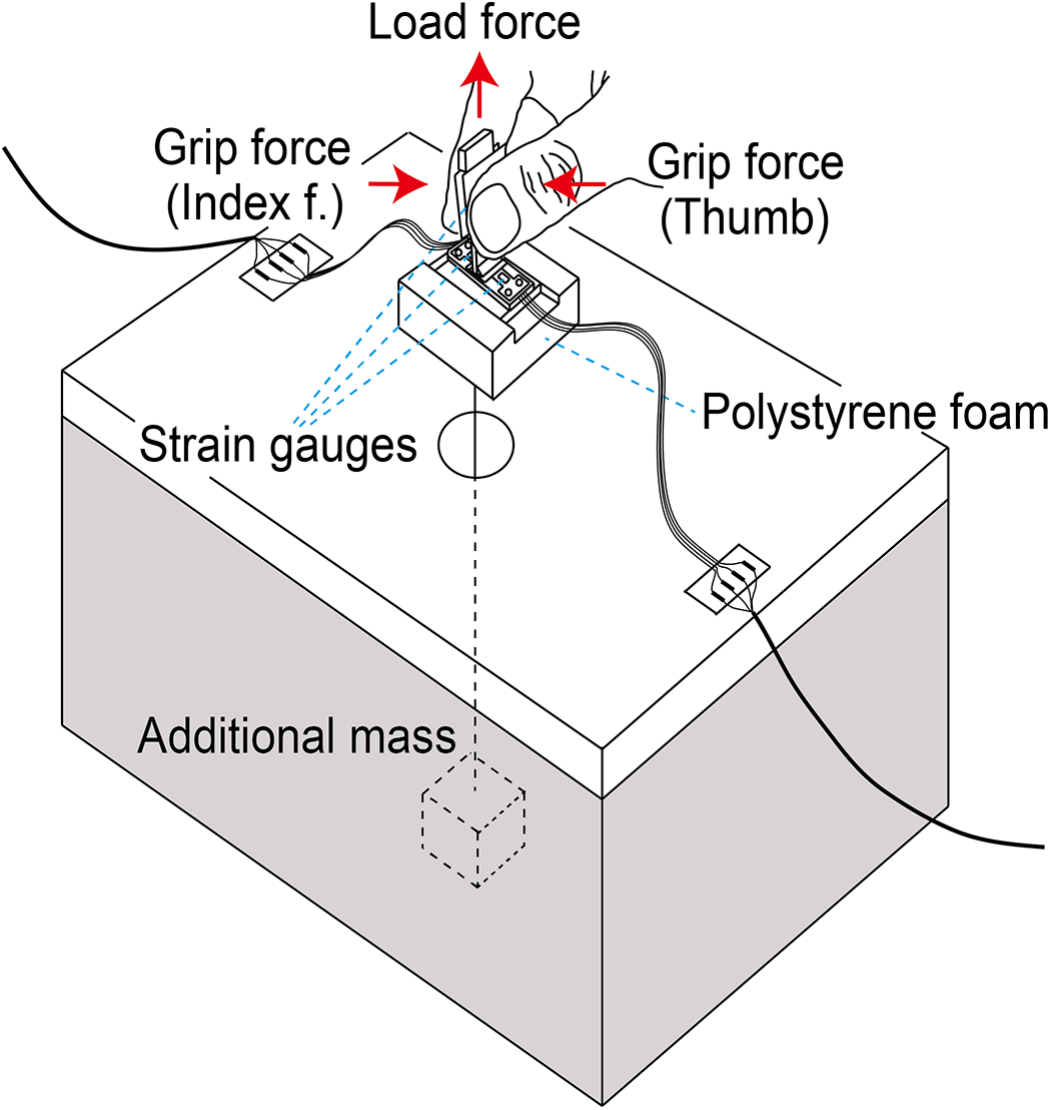
The light grip apparatus and the experimental table. The apparatus contained three sensors to measure grip and load forces.

The grip apparatus shown in Fig 1 consisted of three lightweight force transducers, thin flexible electric wires, and a polystyrene cushion form at the bottom. It had a mass of 6 g and a height of 60 mm. It was a size- and weight-reduced version of the apparatus introduced in earlier studies (see details shown in [9], [10], [12]). The design for the arrangement of the force transducers was thus basically the same as those of the earlier studies. Briefly, the grip and load force transducers had a double-end supported beam structure, and each of those had 4 strain gauges to form a resistive Wheatstone bridge circuit. The force sensing beams were precision-machine-cut from a 0.12-mm-thick steel plate (length: 32 mm). The supports for the beams were pieces of small block cut from 3-mm-thick hard aircraft-grade (AG) plywood, which were screwed using microscrews, and also glued firmly to both ends of the beam. For the grip force transducer, a pair of grip surface plates made of 1.5-mm-thick hard AG plywood were also firmly glued to the center of the beams. The grip force transducers allowed the separate measurement of grip (normal) forces generated by the thumb and index finger when gripping the apparatus. The distance between the two grip surfaces was 7.2 mm. For the load (lifting) force transducer, the beam with end supports was firmly fixed to a base made of 2.5-mm-thick AG plywood with a vertical AG plywood bar for reinforcement against a bending moment.

There were 8 electric wires from the grip apparatus, which consisted of 2 for electrical power input and 6 for strain gauge signal output from the 3 force transducers. To balance the mechanical effect of the wires, they were divided into both sides of the bottom of the grip apparatus. Two types of electric wire were used in this study. The first type, a 20-cm extremely fine (diameter: 0.11 mm) insulated copper strain gauge lead cable, provided an initial connection from the grip apparatus to relay terminals fixed on both sides of the experimental table (Fig 1). The second type was ordinary shield electric wires for strain gauges, which provided a connection between the relay terminals and the Kyowa DPM-601 strain amplifiers. The first type of cable was almost weightless (>0.01 g) and flexible, so that the effect of wire elasticity could be minimized. As a preliminary study, using 3 subjects, we tested the effect of mechanical interference caused by the first type of cable on low grip force while lifting the grip apparatus under selected low-mass conditions (6 g, 10 g, and 22 g) with a sandpaper surface. To this end, we removed 4 wires from among the original 8 wires while keeping one grip force transducer active. Grip force data from this force transducer were compared for each subject who performed a task of lift-to-hold for 10 sec before and after removing the wires. The mean static grip force computed from data from 20 trials did not differ significantly between the 4- and 8-wire conditions in each mass condition for each subject, suggesting that the mechanical effect of the electric wires connecting the apparatus and the relay terminals on the table would be quite small or negligible. Static loading tests on each sensor revealed linearity up to 3 N with force resolution of 0.001 N.

During the experiment, different levels of apparatus mass were created by hanging an additional mass from the bottom of the apparatus using a string. The grip apparatus was always placed on an experimental table (height = 45 cm), which had a 4-cm-diameter hole in the center, and through this hole, the additional mass was hung. The bottom of the table was covered with pieces of wooden panel to hide the added mass from the subject’s view. Fourteen apparatus mass conditions: 6 (no additional mass), 8, 10, 14, 22, 30, 40, 50, 70, 90, 110, 130, 150, and 200 g, were tested in this study. In order to modulate the frictional conditions at the grip surface, a thin sheet of sandpaper (No. 240) or a piece of smooth rayon cloth was attached on the grip surface using thin double-sided adhesive tape. The amplified force transducer output signals were digitized using a personal computer via 12-bit A/D converter sampling at 600 Hz for each channel.

### Experimental tasks and procedure box

#### Lift-hold task

All 17 subjects performed this task. Each subject prepared his/her right hand by placing it near the apparatus, and waited for a short computer-generated beep indicating the onset of gripping and lifting of the apparatus by a precision grip using the index finger and the thumb. The apparatus was lifted at the subject’s self-determined lifting speed to a height of about 5 cm above the top plate of the table, where it was held stationary until a second beep coming 11 sec after the first one. The subject then dropped the apparatus on the table by slowly separating the fingers. Each subject performed 8 trials for each of the 14 mass conditions for each of the two surfaces, and thus provided trial data consisting of a total of 224 values. For each subject, the mass conditions were presented randomly while the surface was kept constant. The surface order was randomized for each subject.

Prior to the experiment, each subject washed his or her hands with soap and tap water. The subject was then seated in a comfortable and stable chair in front of the experimental table on which the grip apparatus was placed. After careful verbal explanation by the experimenter about the procedure, the subject observed 3–5 demonstration trials performed by the experimenter, and performed the task with different mass and surface conditions until feeling comfortable in terms of the ability to perform the task. Because sliding-to-dropping of the object slowly at the end of each task was difficult for some subjects, this process was repeatedly practiced until the subject was confident about their ability to perform it. Prior to the data collection for a given mass condition for a given surface, the subject again practiced the lift-hold task three times in order to experience the new condition. This was performed systematically in order to minimize the aftereffects of the prior condition on the current condition. Each subject had a break of several minutes between different mass conditions to prevent fatigue.

#### Weight discrimination task

On a separate day from the lift-hold task, 9 of the subjects underwent a weight discrimination task using two light grip objects (mass = 4 g) having the same dimensions as the grip force measurement apparatus. No force sensors were installed in this object. A piece of string 15 cm long with a hook was attached at the bottom of this object in order to hang an additional mass. The grip surfaces were covered with either sandpaper (No. 240) or rayon material. The two objects were placed 10 cm apart and side-by-side on an experimental table with two small holes. The bottom of the table was covered with wooden panels to hide the added mass from the subject’s view. The object placed on the right side always had the standard mass, and that on the left side had the test mass. Four sets of test mass, with each set comprising 9 levels of test mass and one standard mass, were used to find a just noticeable difference (JND) in object mass within each set. The standard mass objects weighed 10, 24, 48, and 112 g, and the weights of their corresponding test masses ranged from 5 to 15 g (1.25-g intervals), from 15 to 33 g (2.25-g intervals), from 35 to 61 g (3.25-g intervals), and from 94 to 130 g (4.5-g intervals), respectively.

In a similar manner to the grip force measurement tasks, the subject lifted and held the right-side object stationary for about 7 sec above the table and then replaced it on the table. Following a 4-sec rest, the subject was asked to lift and hold the left-side object for about 7 sec, and to replace it on the table. The subject then reported verbally to the experimenter whether the second object was lighter or heavier than the first one. For each mass set, this forced weight discrimination was performed in 63 trials in which the test mass presented for each trial was pseudo-randomly changed. This provided 7 values of response data for each test mass-surface condition per subject. The orders of the mass set and surface presentation were randomized for each subject. A total of 504 trials (63 trials x 4 mass sets x 2 surfaces) were thus performed by each subject. To reduce the effect of mental and physical fatigue, two separate sessions were used for testing different surface conditions. In addition, an adequate rest period (3 min or more) was given before testing a new mass set.

### Data analysis and statistical tests

All trial records of the grip and load force data from the lift-hold task were processed off-line to compute six parameters evaluating grip and load force control. During this process, grip forces from the thumb and index finger were averaged [9,12]. The parameters evaluated were static grip force, coefficient of variation (CV) of static grip force, grip and load force ratio (a GF/LF ratio), slip force, safety margin force, safety margin force relative to the static grip force (relative safety margin), and a coefficient of static friction. The static grip force was calculated by averaging the grip force and corresponding load force over the period between 4 and 8 sec from the onset of the initial beep. During the same period, the standard deviation (SD) of the static grip force was also computed to evaluate static grip force fluctuations (within-trial variability). The CV of static grip force was then computed by the SD divided by the static grip force as a measure of standardized grip force variability. The GF/LF ratio was calculated by dividing the static grip force by the mean of the load force between 4 and 8 sec from the initial beep.

The slip force was the minimum grip force required to prevent slippage, and it was measured at the moment when the apparatus began to slip. To facilitate identification of the slipping moment, we computed the load force rate by first digitally filtering the load force data using a low-pass method (4th-order Butterworth) at a cut-off frequency of 30 Hz, and subsequently calculating the differences in load force between consecutive samples. We also computed the SD of the static load force during the period between 4 and 8 sec from the initial beep to facilitate the determination of slip events. A slip event was characterized by a marked fall in the load force as well as its rate. For each trial, the slipping moment was approximated first visually from the load force and load force rate records displayed on the PC screen, and secondly, for the approximated period, the slip event was defined algorithmically when the load force dropped below the threshold value of -2 SD. The corresponding slipping moment was further defined from the load force rate data when the value became negative. In the light mass conditions (< 22 g), there were sometimes multiple slip-stick events. In such cases, the earliest defined slip event was adopted. The identification of the slipping moment was difficult for some trial data owing to numerous excessively small slips, possibly caused by microslips, and small object tilting. Of 1904 trials (17 subjects x 14 masses x 8 trials) for each grip surface, the numbers of trials for which a clear slip moment could not be ensured were 179 (9.4%) for sandpaper and 198 (10.4%) for rayon. However, all subjects provided at least 6 trials of slip force data for each of the mass and surface conditions. The safety margin was then the difference between the static grip force and the slip force. The static friction coefficient was calculated as the ratio of slip force to load force at the moment the finger slipped.

For each of the grip force parameters, the mean of 8 trials was computed for each mass-surface combination for each subject, which was used for the subsequent statistical tests. Two-way ANOVA with repeated measures was performed to test the effects of mass and surface on the GF/LF ratio, relative safety margin, coefficient of static friction, and CV of static grip force. A post hoc test was carried out by the Tukey multiple comparison method. Statistical significance was accepted at P<0.05.

### Additional measurements

In order to facilitate understanding of light object prehension, three additional measurements were also performed for all 17 subjects. First, grip force-related changes in the contact area of the finger skin were estimated using the right thumb and index finger. A transparent acrylic plate (130 x 130 x 8 mm) having force sensors at each of its four corners and a fluorescent light to emboss the plate contacting skin in a dark environment were used in this measurement. The force transducers were firmly fixed to a metal plate that had a circular hole (diameter: 40 mm) in the center to view the skin contacting the acrylic plate. The metal plate was fixed vertically on a test table so that the subject could press his/her finger on the acrylic plate in a manner similar to gripping the test apparatus. The skin contacting the plate was recorded using a high-resolution digital video camera sampling at 30 Hz. Recorded video images following contrast processing were obtained using Otsu’s clustering-based image threshold technique [47,48], and the pixel-based computation of the area of contact (the white area) was subsequently performed to compute the apparent contact area [49]. On the basis of externally triggered signals during the measurement, the force data sampling at 120 Hz was time-matched with the area data. Second, the stress-strain relationship of the fingertip during gripping of an object was assessed for each subject. The same grip force transducer used for the grip apparatus was firmly attached to a vertical plate fixed on the test table, and the subject’s right hand/forearm rested and height-adjusted on a table gripped the force sensor using the right thumb or index finger while a slide-type friction-free precision position sensor (Kyowa Ltd., DLT-50AS) was attached to the nail of the testing finger to measure the fingernail position relative to the grip surface. These position data were used to estimate finger skin indentation during grip force application. The gripping force and finger position data were simultaneously recorded upon PC sampling at 600 Hz.

## Results

### Lift-hold tasks

**Fig 2:**
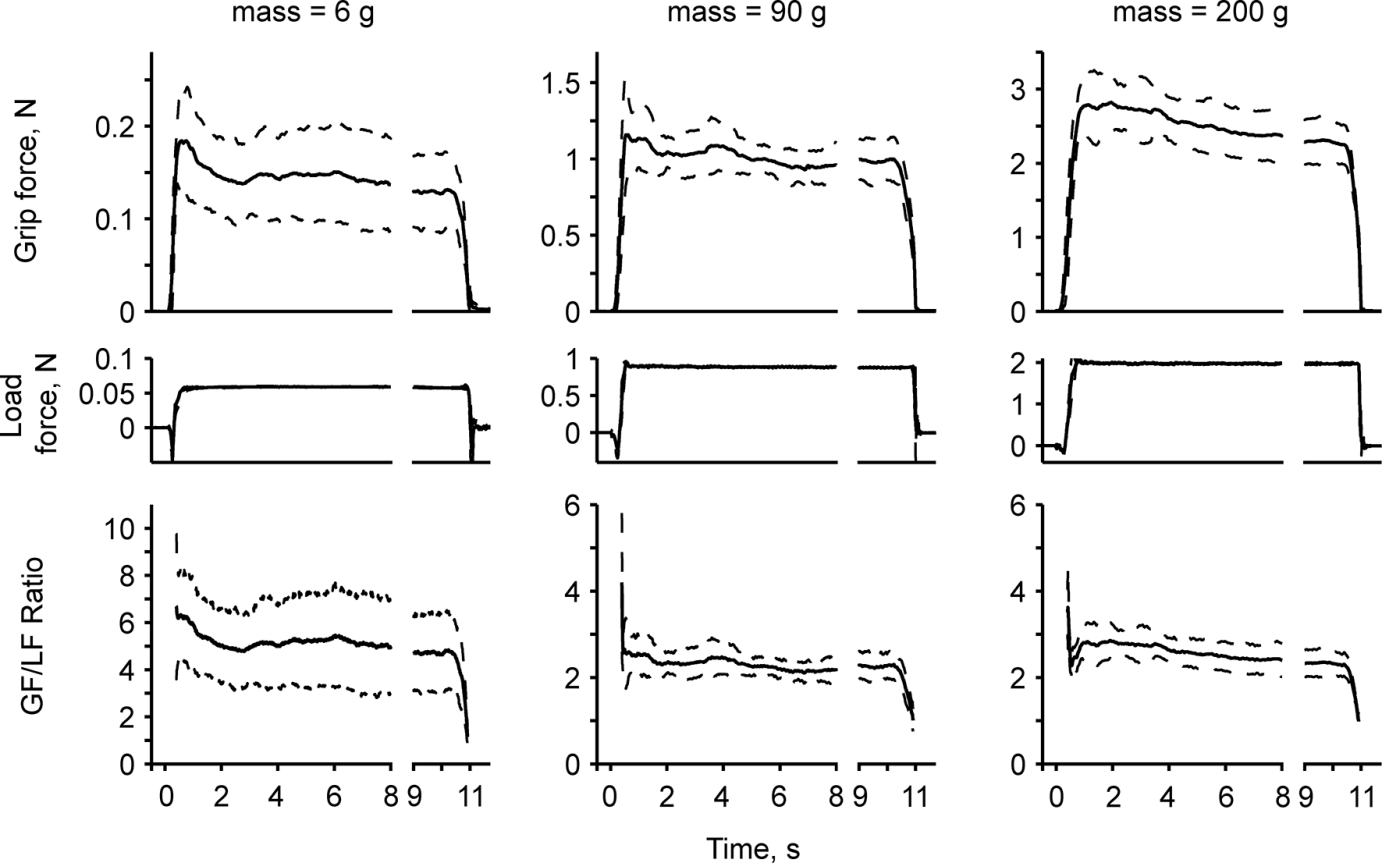
Typical force-time history curves of grip and load forces during the lift-hold task performed by one subject. The data are the mean (solid lines) ± standard deviation (dashed lines) of 8 trials for the 6, 90, and 200 g mass conditions with the rayon grip surface.

Fig 2 shows typical examples of mean grip and load forces as a function of time in one subject during the lift-hold task with the apparatus set to 6, 90, and 200 g under the rayon surface conditions. The grip and load forces generated were fairly smooth from the grip onset, and formed a small peak shortly after the apparatus had been lifted from the table. The grip force then relatively smoothly transitioned to a holding phase. A slight decrease in the magnitude of grip force was noted in some of the heavier mass conditions until the moment of release at the end of the 10-sec holding phase (see the 200-g data in Fig 2). These force patterns were fairly common to all subjects.

**Fig 3:**
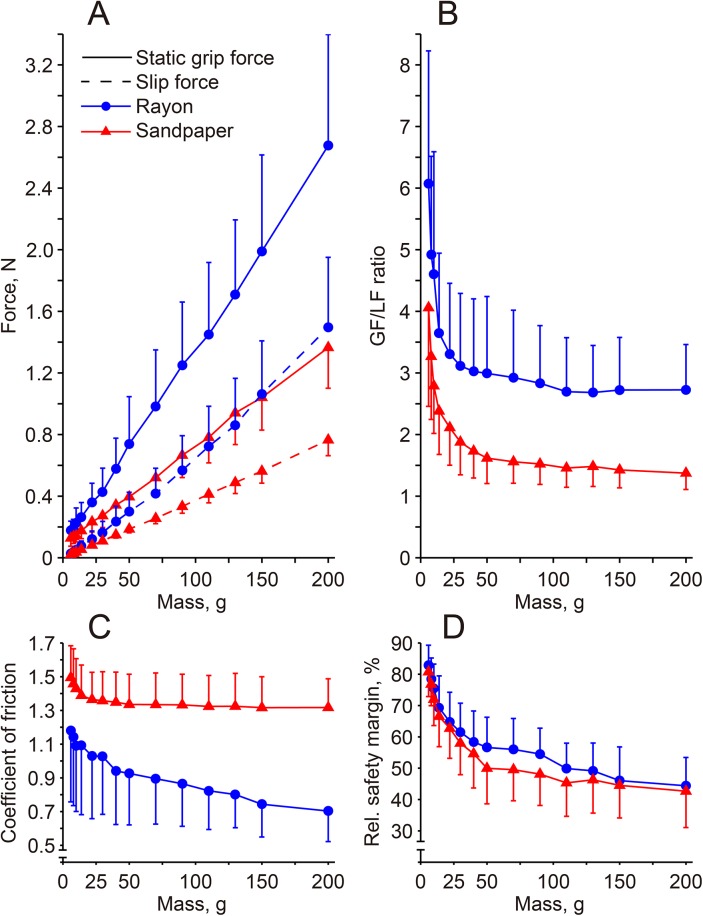
Changes in the static grip force and slip force (A), grip-to-load force (GF/LF) ratio (B), coefficient of friction (C), and relative safety margin (D) in relation to object mass for each surface condition. The data represent the mean and standard deviation (vertical bars) values of 17 subjects.

In Fig 3A, the mean values of static and slip forces for all subjects in relation to the apparatus mass for each surface condition are shown. The mean static force ranged from 0.18 N while holding the 6-g object to 2.68 N for the 200-g apparatus for the rayon surface, and the corresponding values for the sandpaper surface were 0.13 to 1.37 N, respectively. These changes occurred approximately in proportion to the object mass for both surfaces, which agrees with the findings of Westling and Johansson [12], who examined the range of object mass from 100 to 1 kg. Differences in static grip force and slip force increased with object mass, so the subjects retained a greater grip force as a margin against slippage for the objects with a heavier mass (Fig 3A). The subjects also provided a greater safety margin force when holding the apparatus with the rayon surface than that with the sandpaper surface. For the 6-g mass, for example, the mean margin force was 0.11 N with the sandpaper surface, while it was 0.15 N with the rayon surface. The margin forces for both surfaces increased almost linearly with an increase in mass of the apparatus, and while holding the 200-g mass, the margin force increased to 0.61 N for the sandpaper surface and 1.19 N for the rayon surface. ANOVA confirmed the main effects of surface (F_1, 247_ = 41.7, p < 0.0001) as well as mass (F_13, 247_ = 104.9, p < 0.0001) on the grip force safety margin.

Grip and load forces were standardized by the value of the apparatus mass lifted (Fig 2). It became evident that the standardized grip force curve of the 6-g condition was much greater than those of the 90- and 200-g conditions. The difference in grip force between 90 g and 200 g, on the other hand, was less clear. In Fig 3B, the mean value of the GF/LF ratio for all subjects is shown. The ratios for both surface conditions were highest with the lowest mass condition, which decreased non-linearly with apparatus mass. The ratios were fairly constant above an apparatus mass of 30 g for both surface conditions. ANOVA revealed significant main effects of mass (F_13, 247_ = 63.6, p < 0.0001) and surface (F_1, 247_ = 35.1, p < 0.0001), with no interaction between these variables. In order to assess the level of lifted mass that caused the upper shift of the GF/LF ratio from a constant value, multiple comparison was performed using the value of the 200-g condition as a reference. This multiple comparison revealed that the ratios for object mass of 14 g or below for the rayon surface and 22 g or below for the sandpaper surface were significantly greater than those of the reference value of the corresponding surface.

The coefficient of static friction was highest with the 6-g condition, and it dropped non-linearly with increasing apparatus mass for both surfaces (Fig 3C). This non-linear increase of the coefficient of static friction towards a lighter tangential load (<0.5 N) has also been reported elsewhere [44,45]. The mean values of the coefficient of static friction for the sandpaper surface were 1.49 for the 6-g object and 1.32 for the 200-g object, and those for the corresponding object mass with a rayon surface were 1.18 and 0.71, respectively. These friction coefficient values for the 200-g object with sandpaper and rayon surfaces are comparable to those reported elsewhere [4,6,12]. As expected, ANOVA revealed significant mass (F_13, 247_ = 30.9, p < 0.0001) and surface effects (F_1, 247_ = 33.9, p < 0.0001) on the friction coefficient.

The relative safety margin, the proportion of the safety margin force to the static grip force, was around 40% to 50% when holding an apparatus weighing 100 g or more (Fig 3D), which is in accordance with the range of the relative margin reported in previous studies [4,12]. The values were clearly increased when holding lighter masses, and indeed they reached around 80% when holding a 6-g mass for both surface conditions. The changes in the relative safety margin with the mass were non-linear. ANOVA revealed a significant main effect of mass (F_13, 247_ = 157.1, p < 0.0001), with no surface and surface x mass interaction effects. A Tukey post hoc test revealed that the values for objects weighing less than 90 g for the rayon surface and less than 70 g for the sandpaper surface were significantly greater than those for the 200-g mass (p < 0.01).

### Static grip force fluctuations

**Fig 4:**
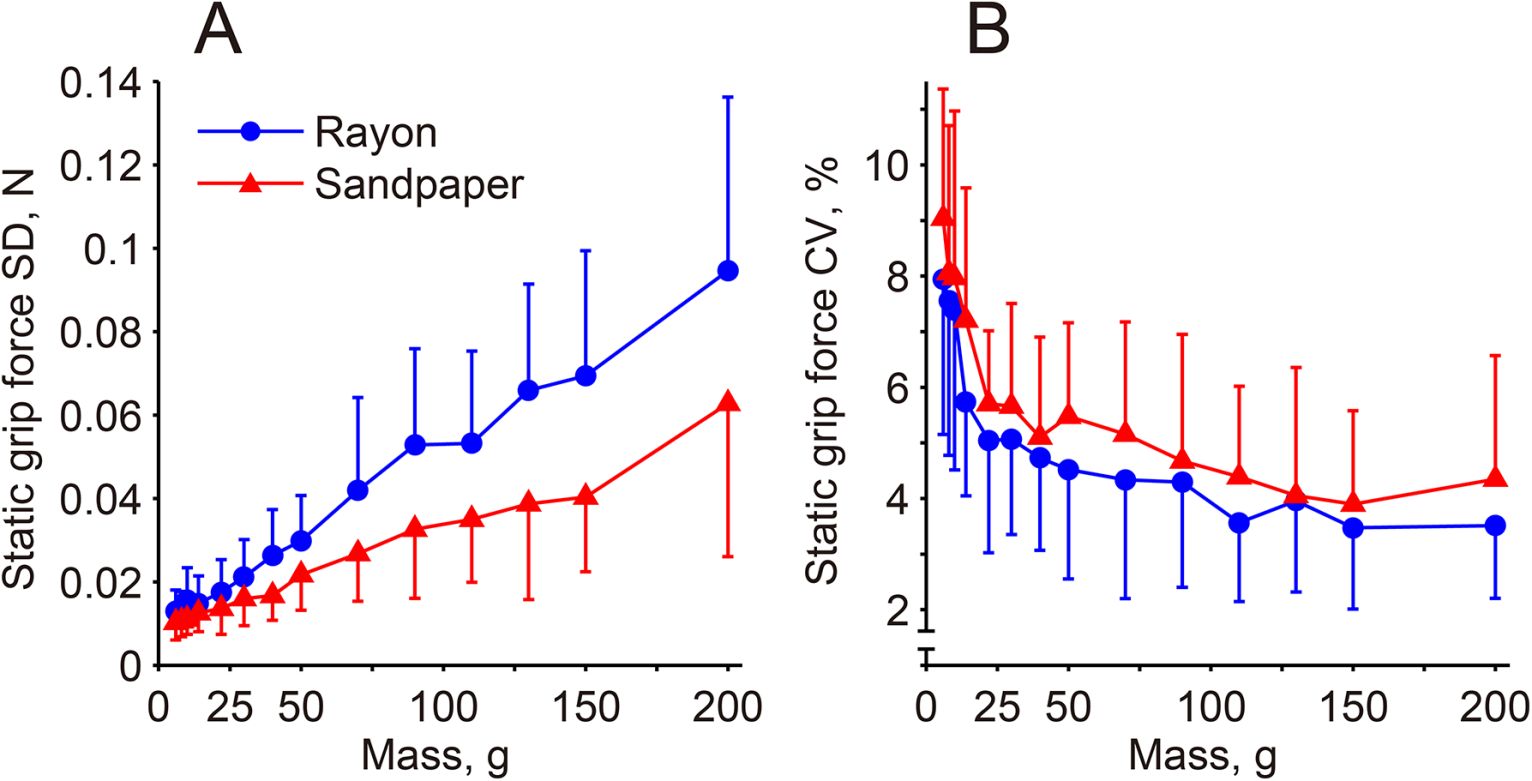
Within-trial variability of static grip force expressed by standard deviation (A) and coefficient of variation (B). The data represent the mean and standard deviation (vertical bars) values of 17 subjects.

The mean value of within-trial variability (SD) of static grip force for all subjects was increased from 0.013 N with the 6-g mass to 0.095 N with the 200-g mass for the rayon surface, and the corresponding values for the sandpaper surface were 0.011 N and 0.063 N, respectively. Their increases with object mass were approximately linear (Fig 4A). The standardized grip force variability (CV of the static grip force), on the other hand, had a non-linear relationship with apparatus mass, while showing the highest mean for the 6-g condition for both surfaces (Fig 4B). ANOVA followed by a post hoc comparison with the 200-g data confirmed that the differences in the mean values of a 30-g mass or lighter for the rayon surface and a 14-g mass or lighter for the sandpaper surface were significant, clearly indicating that force fluctuations were elevated when holding objects in the lower end of the mass range examined.

### Weight discrimination task

**Fig 5:**
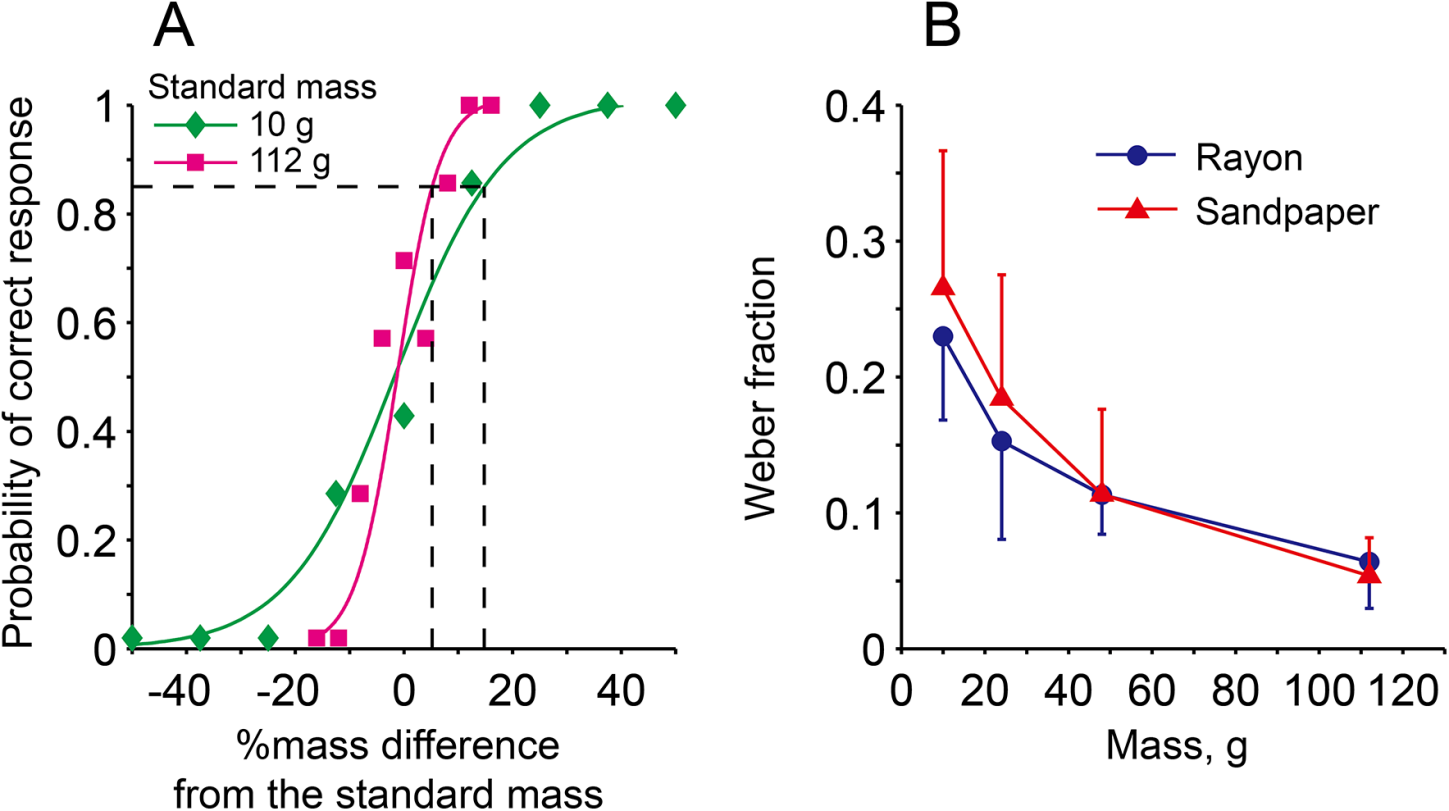
A typical example of the mean probability of correct response in relation to percent difference in test mass from the standard masses of 10 and 112 g for the rayon surface in one subject (A). Logistic function curves were fitted to examine their relationship. Changes in mean Weber's fraction with test mass for all subjects for the rayon and sandpaper surfaces (B). The vertical bars indicate the standard deviation values.

For the individual weight discrimination data for each surface condition, the mean probability of correct response was computed using 7 trial data at each of the 9 test-mass levels. The 9 mean probability values were then plotted in relation to the value of %mass difference from the test (standard) mass. A logistic function was fitted to these individual data to estimate the value of JND defined as the stimulus magnitude of the comparison at which the proportion of correctly discriminated heavier test objects equal to 0.85 [50]. Typical examples of the JND estimated for one subject with the 10-g and 112-g test sets under the rayon surface condition are shown in Fig 5A. Note that the fitted curve for the 112-g test set had a steeper slope than that of the 10-g test set, resulting in a greater Weber fraction value for the 10-g test set compared to the 112-g test set.

Fig 5B shows the mean JND values for the 9 subjects computed for all four mass sets under the rayon and sandpaper surface conditions. For both surfaces, the JND corresponded to a Weber fraction value of 0.06 (6% of the standard mass) for the 112-g test set. The JND was clearly elevated with a decrease in test mass, reaching 0.29 (29% of the standard mass) for the 10-g set, close to a fivefold increase from that for the 112-g set. ANOVA with a multiple comparison confirmed significant differences in the JND between 10-g and the other three mass conditions, and between 24 g and 112 g. The ability to discriminate heaviness held by the fingers was thus not proportional to the object mass. These findings confirmed that a small test object of equal dimensions to the instrumented grip apparatus produced results of weight discrimination similar to earlier observations by Oberlin [30], and Ross and Reschke [51], who reported that Weber-fraction-related heaviness perception rose largely for objects with a small mass below 50 g.

### Mechanical properties of the finger pad

**Fig 6:**
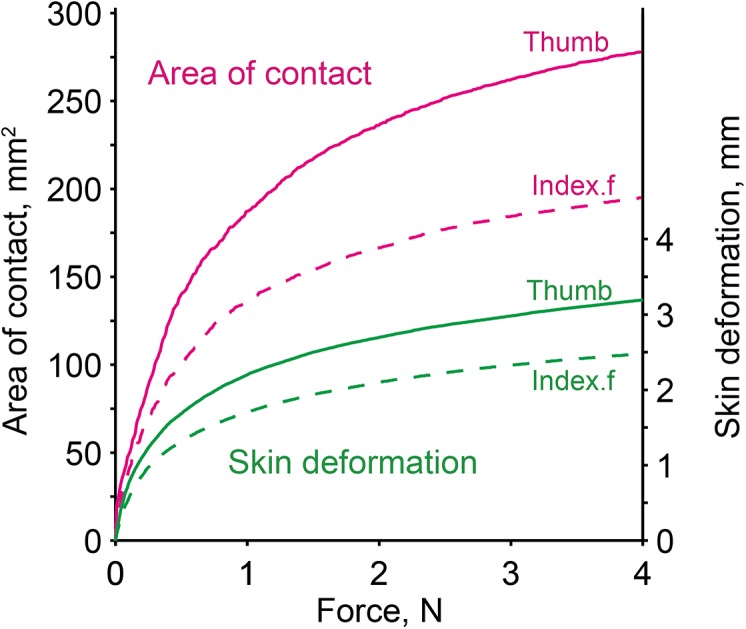
Mean finger-surface contact areas and finger pad skin deformation for the thumb and index finger for all subjects in relation to varied magnitude of grip force applied.

Fig 6 shows grip-force-related changes in the mean value of apparent surface contact area by the index finger and thumb for all 17 subjects. The area sharply decreased when the applied grip force was below 1 N, and the decrease was even sharper below a grip force of 0.5 N. Curve fitting on the average apparent contact area (Area) as a function of grip force (GF) was performed using a power function (Area = *a*GF^*b*^). Grip force between 0.01 N and 4 N gave values of *a* and *b* for the index finger of 122.9 mm^2^/N, and 0.409 (r^2^ = 0.96) and those for the thumb of 169.8 mm^2^/N and 0.436 (r^2^ = 0.96), respectively. These values are close to the average values provided in a previous study [49].

In Fig 6, the finger pad indentation and grip force relationship is also given. Similar to the changes in contact area, the pads for both the thumb and the index finger were largely deformed upon the application of grip force up to 0.5 N, and the increase in deformation gradually tapered with larger force application. Curve fitting with a power function (indentation = *c*GF^*d*^) on the mean data provided values of c and d for the index finger of 1.57 m/N and 0.40 (r^2^ = 0.94), and for the thumb of 2.02 m/N and 0.40 (r^2^ = 0.95), respectively.

## Discussion

Westling and Johansson [12] previously showed that, while holding a movable object using a precision grip, the generated grip force was proportionally modulated in relation to the object mass ranging from 100 g to 1 kg, so that the GF/LF ratio could be kept nearly constant for a given grip surface. The present results basically support their findings, and further suggest that the same relationship could be retained down to the lower range of object mass (>30 g). On the other hand, for objects lighter than 30 g, the healthy adults employed a progressively higher GF/LF ratio. The ratio for the 6-g object, namely, the lowest mass tested in this study, was more than double those upon holding a mass of 100 g or above for both the sandpaper and the rayon surfaces. A clear increase in the GF/LF ratio while holding an object was also reported by researchers who performed precision grip experiments while their healthy young subjects were experiencing microgravity induced by parabolic flights [25,26]. They found that a grip force from 0.9 N to 0.4 N as a pure grip force safety margin was present while holding a weightless object.

Because the grip force for holding an object having a certain mass can be made up of the components of slip force and safety margin, either or both of these could be increased to provide a high GF/LF ratio. The evaluation of each of these in the present study confirmed that the increase of the safety margin was exclusively responsible for the raised GF/LF ratio for the lifting of very low-mass objects (< 30 g). It was also confirmed that the slip force did not increase, but it instead progressively decreased towards the lower end of object mass. An increase of the GF/LF ratio by an enlarged safety margin is discussed in association with the hypotheses posed in the introduction.

### Decreased tactile sensibility for holding a light object

Studies using local anesthesia of grasping digits [12,23,52–54] have repeatedly shown that cutaneous sensory inflow plays a crucial role for the CNS in properly adjusting the GF/LF ratio during lifting and holding of an object. Loss of cutaneous sensation commonly causes disability of proper surface friction and load force adaptation, resulting in a high GF/LF ratio with an excessively elevated margin of grip force [52]. Experiments using gloves with different thicknesses of material [10] and compression of the median nerve at different pressure levels [20] have further demonstrated that graded hypoesthesia can lead to a corresponding increase in the magnitude of grip force used. It is thus possible to conjecture that graded increases in both the GF/LF ratio and the relative safety margin with a lighter object are attributable to a corresponding decrease in available cutaneous sensory input. A crucial factor determining the magnitude of tactile sensory input can be the number or population of activated mechanoreceptors at the gripping site. Four types of cutaneous mechanoreceptor (FAI, FAII, SAI, and SAII) innervating the glabrous skin of the human hand are known to convey sensory signals to the CNS for the control of grip force [55,56]. FA types have strong sensitivity to dynamic stimuli including slip-induced skin vibration. SA types are sensitive to sustained skin indentation and stretch. Among these four, FAI and SAI units (some FAII units as well) are known to play an important role in providing afferent signals to the CNS to maintain static grip force at an intended low level. The primary afferent signals are commonly from microslips localized to the peripheral zone (10–30%) of the contact area, which appear when static grip force with a small safety margin is further decreased [1,13,57]. When the local slip zone exceeds about three-quarters of the contact area, an overall slip commonly occurs, which gives a strong burst of afferent signals to trigger a rapid increase in grip force [56,58]. The area of contact and skin indentation data suggest that the available population of cutaneous units possibly activated decreases sharply below a grip force of 0.4 N (Fig 6). The population signaling the localized slips would then be even smaller. A grip force of 0.4 N corresponded to the static grip force observed while holding a 30-g object with a rayon surface, suggesting that the population of cutaneous receptors activated below this may prompt a feeling of insecurity regarding sensory feedback required for stable grasping. The results from the present weight discrimination test showing a clearly higher Weber fraction with a lighter object seem to support this.

### Central control for lifting and holding an object with a low mass

Centrally organized motor commands must have contributed to the elevated GF/LF ratio along with the high relative safety margin in the current low mass range (<30 g). Behavioral studies have repeatedly shown that lifting of an object is basically performed by the prediction of its mechanical properties, including mass, surface friction, size, and shape [17,18,22,59]. The appearance of the object and sensory memories acquired through previous lifts are essential for the CNS to make an accurate prediction on the generation of grip force at object-lift onset. A smooth transition from lifting to stable holding of the gripped object also takes place with accurate anticipation of an object’s properties [11,18]. To facilitate the sensory memory-based performance in the present study, the data were always collected from repeated lifts with objects having the same properties. As expected, we observed the profiles of grip and load forces with fairly smooth increases without forming either an excessively high peak or multiple peaks during the loading phase, and a smooth transition to a stable level for static holding across all object mass conditions (Fig 2). On the other hand, we also observed a common high GF/LF ratio during the phase of load force increase for the light objects, which poses questions first about the effect of finger-surface frictional change with small grip force application, second about the goodness of the sensory memory used, and third about the complexity of fine fingertip force control.

Firstly, a priori knowledge about the current frictional state at the finger-surface interface plays a dominant role in the setting of the GF/LF ratio during object lifting [17]. Predictive grip force production with a higher GF/LF ratio has been found during lifting and holding of a more slippery object [9,56]. This was basically true in the present study, in which the force ratio for the rayon was set higher during lifting and holding than that for the sandpaper at all object masses. By contrast, this does not seem to be the case for the effect of the mass-related frictional change. Slip force assessed in our subjects indicated that the friction clearly increased at the lower mass range (<30 g) compared with that for the heavy range (> 100 g), and the GF/LF ratio followed a similar pattern. Therefore, the possibility of an effect of friction on the increased force ratio during low-mass-object lifting is unlikely.

This leads to the second possibility of doubt about the goodness of the sensory memory. As discussed above, when manipulating a low-mass object, sensory afferents from the fingertips were most likely insufficient. The results of the psychophysical test at the low mass range also indicated the difficulty of accurate mass perception acquired from both proprioceptive and cutaneous sensors. With such limited tactile sensory inflow, sensory memory of the current object properties would also be formed less accurately and affect the goodness of memory-based proper feed-forward scaling of the grip force. The increased GF/LF ratio at lifting onset and during the holding phase can be considered as a compensatory and learned strategy employed by individuals with declined cutaneous sensory function, such as observed in carpal tunnel syndrome patients [34,35] and the elderly [7,9]. We conjecture that a similar strategy was adopted by our healthy adults when manipulating regular items with a small mass while facing a trade-off between the economy of some extra muscular work and a secure feeling of grasp stability with increased tactile sensory inflow and a greater contact area.

The third possibility is difficulty in producing a sustained grip force at an intended low level due to increased variability of motor unit discharge in the muscles used [41,43,60]. The proportion of internal noise in the common neural drive to moto-neurons of target muscles is known to be increased at low contraction intensities in some muscles, including those in the hand [61,62]. This commonly leads to a greater proportion of fluctuations (CV values) in generated force, causing a decline in optimal control of sensori-motor performance due to a decreased signal-to-noise ratio [63]. Using the CV values of isometric force, studies have indeed demonstrated that force fluctuations at weak levels (2–4% of maximum voluntary contraction) were apparently greater than those at moderate levels (12–50%) [39,42]. Our static grip force CV values followed a similar pattern; the value with 6-g mass was highest, which decreased non-linearly with increased object mass.

Could the magnitude of force fluctuations observed when holding low-mass objects actually threaten grasp stability? To test this, hypothetical (non-elevated) static grip force was estimated for each of the low-mass conditions (<30 g) based on the assumption that the subjects used a GF/LF ratio similar to that for larger-mass conditions (110–200 g) for the low-mass conditions. The mean ratios of the heavier conditions were 1.44 for the sandpaper and 2.71 for the rayon, which would provide estimated grip forces of 0.08 and 0.04 N for these surfaces when holding the 6-g object, for example. Subtracting slip forces of 0.03 N for the rayon and 0.02 N for the sandpaper from these values gives safety margin forces of 0.05 N and 0.02 N for these surfaces. The 95% confidence interval values of static grip force fluctuations for these surfaces were 0.028 N and 0.024 N, respectively. Therefore, unless the subjects voluntarily increased the safety margin during low-mass holding, the fluctuations could have exceeded the lowest limit of the margin, inducing constant reflexive force adjustments or, in the worst case, a loss of the held object, particularly for the sandpaper conditions with a small margin force.

The safety margin forces actually secured by the present subjects for the 6-g mass condition were 0.11 N with the sandpaper and 0.15 N with the rayon. Related to the grip force margin, earlier studies during parabolic flights demonstrated that a pure safety margin assessed from stationary holding of a weightless object with a mass of 471 g with a sandpaper surface was about 1 N [26], and that of an 800-g mass with a brass surface was 0.4 N when the subjects were better adapted to parabolas [25]. Hermsdorfer et al. [26] stated that a margin force of about 1 N would be needed even for a zero-weight object to guard against a sudden increase of the inertial force that may result from perturbations of the stationary arm position. These margin values were thus much larger than our values measured under normal-gravity conditions. This discrepancy most likely arose from motor commands for preparatory control of anticipated inertial force on the object due to the use of apparatuses with 8 to 12 times larger mass in their studies than in ours. The larger safety margin may also be attributed to the microgravity effect, which had not been completely eliminated within the limited period of adaptive training.

Optimal sensori-motor control theories use cost functions to explain how actions are performed [64]. The voluntary grip force for adjusting the safety margin should not be too high to optimize the costs of energy through muscle commands. It could also be applicable to the cost of finely adjusting low levels of grip force to cope with very light objects, which could be quite expensive for central energy. The findings from a functional imaging study by Kuhtz-Buschbeck et al. [14] indeed indicated that a precision grip of an object with deliberate lessening of the safety margin from the ordinary level would demand greater cortical activation in the cortical and subcortical areas, most likely reflecting increased alertness for increased difficulty of grasp stability.

In summary, the instrumented small grip apparatus developed in this study extended our understanding of sensorimotor control of finger forces employed by healthy young adults when lifting and holding objects with an extremely low mass in a normal-gravity environment. It was demonstrated that the GF/LF ratio, a portion of the safety margin in the static grip force, and normalized moment-to-moment static grip force fluctuations were all elevated progressively towards the lower end of the object mass range examined. These occurred irrespective of friction at the grip surface. The findings thus indicate that a strategy of grip force control for holding objects with a smaller mass becomes progressively different from that which was proposed by earlier researchers who used a grip apparatus with a mass greater than 100 g [1,6,12,18]. Test results of weight perception, finger-surface contact area, and skin indentation indicated that feedback signals from the population of cutaneous mechanoreceptors activated while holding an extremely light object were greatly limited. Overall, the present findings suggested that the handling of objects with a very light mass (< 30 g) produced greater reliance on higher cortical function, by which the portion of the safety margin force within the holding grip force was raised in order to reduce the risk of losing the object from the fingers. The declined peripheral sensory input, increased proportions of static grip force fluctuations, and increased care regarding the effect of the inertial component of the load force were considered to be related to the elevated cortical function.

As for future study on light objects, one should examine the rigidity of the apparatus. On a very light and deformable object such as a thin plastic cup, it may be that, while load force (object mass) is too low to be considered as the main factor (or the co-factor with friction) that drives the CNS in forming a precision grip, the deformation of the material can be used instead. In that case, using a very rigid metallic structure could create confusion of the system.

Precision grip is also a focal topic of recent research in rehabilitation. In this regard, an instrumented grip apparatus with a small mass as developed in this study should be useful for the examination of patients with neurological disorders and stroke. These patients commonly incur long-term deficits in sensori-motor function of the hand, often having markedly lowered grip strength with declined fine force control compared with their healthy peers. Finally, the development of an instrumented wireless apparatus, and thus a freely movable device with a small mass, is also urgently needed for the assessment and training of sensori-motor function in neurological and stroke patients.
